# Supplementary material for: The making of a potent L-lactate transport inhibitor
Source: Commun Chem. 2021 Sep 6;4:128. doi: 10.1038/s42004-021-00564-5 (PMC9814091; doi:10.1038/s42004-021-00564-5)
Supplement: Supplementary file 1 — Supplementary Information [file 42004_2021_564_MOESM1_ESM.pdf]

Supporting Information

The making of a potent L-lactate transport inhibitor

Patrick D. Bosshart[1,2], David Kalbermatter[1,3], Sara Bonetti[1,3] & Dimitrios Fotiadis[1]

[1] Institute of Biochemistry and Molecular Medicine, and Swiss National Centre of Competence in Research (NCCR) Trans-Cure, University of Bern, CH-3012 Bern, Switzerland

[2] Present address: leadXpro AG, Park Innovare, CH-5213 Villigen, Switzerland

[3] These authors contributed equally

Corresponding author:
Dimitrios Fotiadis, Ph.D.
Institute of Biochemistry and Molecular Medicine
University of Bern
Bühlstrasse 28
CH-3012 Bern, Switzerland
Tel.: +41-31 631 41 03
E-mail: dimitrios.fotiadis@ibmm.unibe.ch

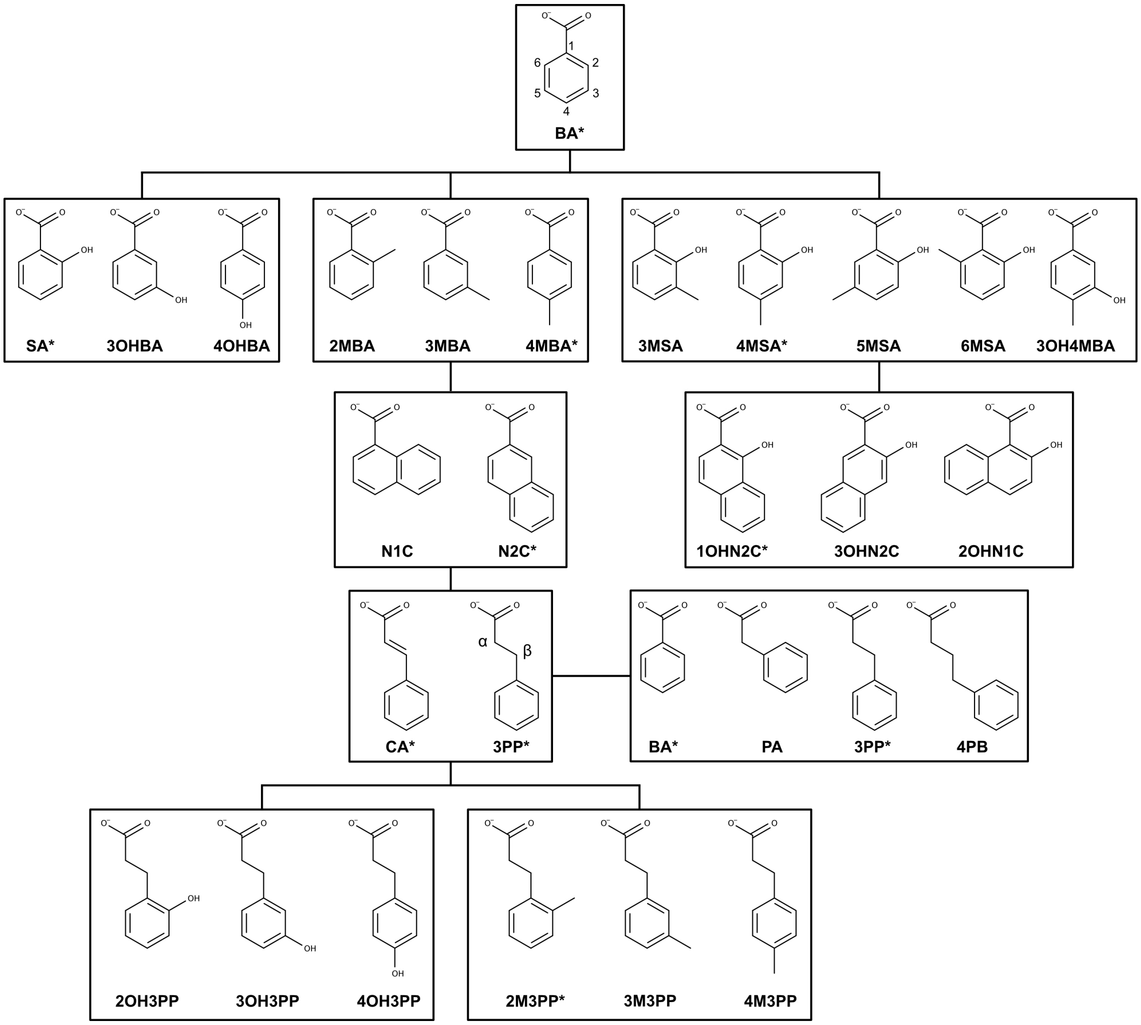

Supplementary Figure 1. Molecular structures of the compounds that were screened for their ability to inhibit SfMCT-mediated [^{14}C]L-lactate transport. Compounds for which K_i values were determined are highlighted by an asterisk. Full names of the shown compounds are: **BA** benzoate, **SA** salicylate, **3OHBA** 3-hydroxy-benzoate, **4OHBA** 4-hydroxy-benzoate, **2MBA** 2-methyl-benzoate, **3MBA** 3-methyl-benzoate, **4MBA** 4-methyl-benzoate, **3MSA** 3-methyl-salicylate, **4MSA** 4-methyl-salicylate, **5MSA** 5-methyl-salicylate, **6MSA** 6-methyl-salicylate, **3OH4MBA** 3-hydroxy-4-methyl-benzoate, **N1C** 1-naphthoate, **N2C** 2-naphthoate, **1OHN2C** 1-hydroxy-2-napthoate, **3OHN2C** 3-hydroxy-2-napthoate, **2OHN1C** 2-hydroxy-1-napthoate, **CA** trans-cinnamate, **3PP** 3-phenylpropionate, **PA** phenylacetate, **4PB** 4-phenylbutyrate, **2OH3PP** 3-(2-hydroxyphenyl)-propionate, **3OH3PP** 3-(3-hydroxyphenyl)-propionate, **4OH3PP** 3-(4-hydroxyphenyl)-propionate, **2M3PP** 3-(2-methylphenyl)-propionate, **3M3PP** 3-(3-methylphenyl)-propionate, **4M3PP** 3-(4-methylphenyl)-propionate.

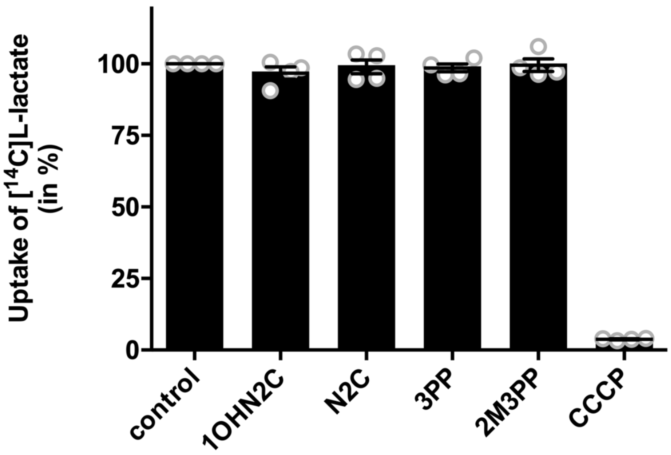

Supplementary Figure 2. Effect of key inhibitors and the protonophore carbonyl cyanide 3-chlorophenylhydrazone (**CCCP**) on [^{14}C]L-lactate transport into *E. coli* MC4100. In the presence of 100 µM of **1OHN2C**, **N2C**, **3PP** or **2M3PP**, no significant reduction in transport activity was observed. In contrast, 100 µM of the protonophore **CCCP** reduced the uptake activity almost completely as previously reported.[1] Therefore, the key inhibitors **1OHN2C**, **N2C**, **3PP** and **2M3PP** do not have a protonophoric effect. Individual data points are shown as open circles.

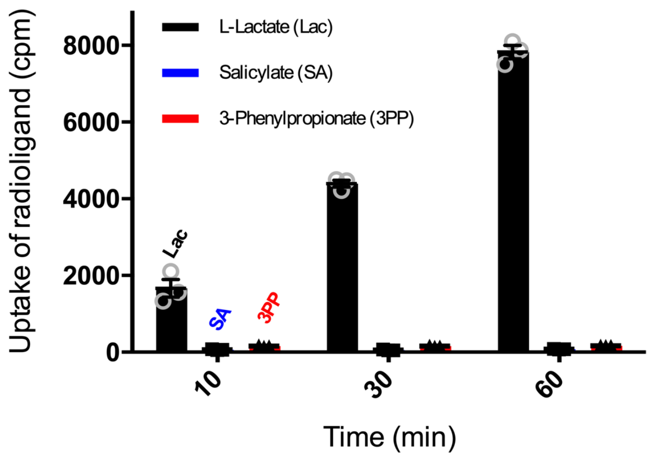

Supplementary Figure 3. Evaluation of selected radiolabeled monocarboxylates (i.e., 0.1 µCi at 40 µM of [^{14}C]L-lactate, [^{14}C]salicylate and [^{14}C]3-phenylpropionate) as potential SfMCT substrates. Data are represented as mean ± SEM from three independent experiments, each in triplicate. If not visible, error bars are smaller than symbols. Individual data points are shown as open circles (Lac), squares (**SA**) and triangles (**3PP**).

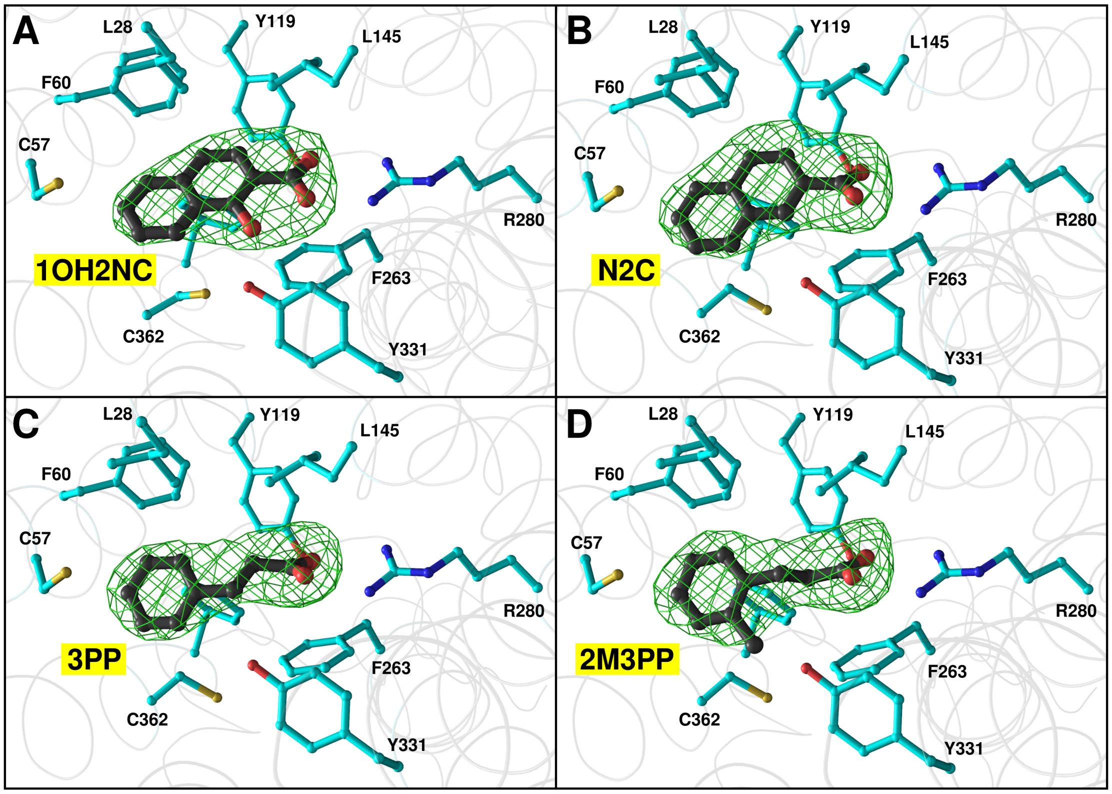

Supplementary Figure 4. OMIT maps for inhibitors bound to SfMCT. The OMIT maps for A) **1OH2NC**, B) **N2C**, C) **3PP** and D) **2M3PP** are contoured at 3.0 σ and colored in green. Specific amino acid residues of the substrate-binding pocket are displayed as ball-and-stick models and highlighted in cyan. PDB IDs of displayed structures are 6ZGR (**1OHN2C**), 6ZGS (**3PP**), 6ZGT (**N2C**) and 6ZGU (**2M3PP**).

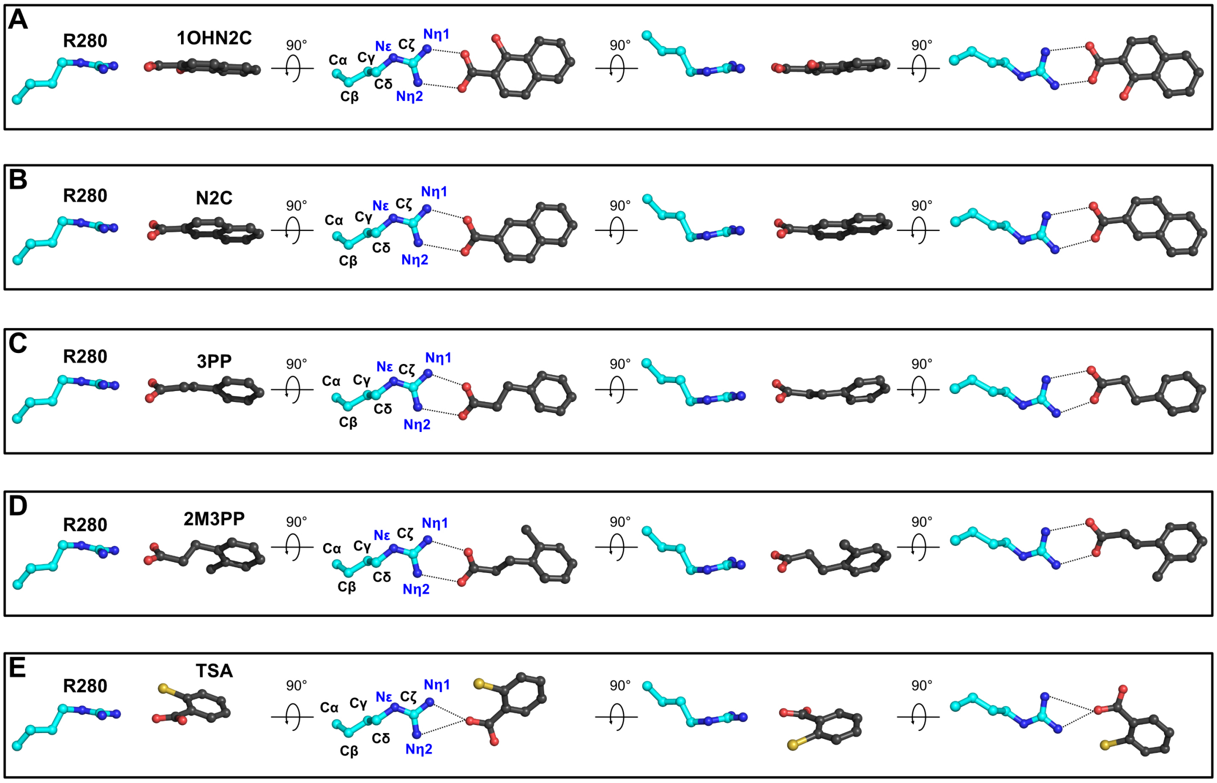

Supplementary Figure 5. Interaction geometries between the guanidinium group of R280 (TM8) and the carboxylate groups of the bound inhibitors (A) **1OHN2C**, B) **N2C**, C) **3PP**, D) **2M3PP** and E) **TSA**).

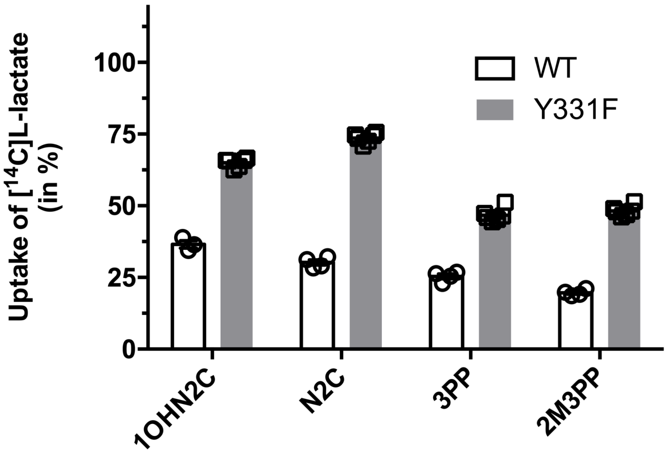

Supplementary Figure 6. Role of the hydroxyl-group of Y331 (TM10) in ligand binding. Wild-type (WT)- and Y331F-mediated [^{14}C]L-lactate transport in the presence of 25 µM **1OHN2C**, 10 µM **N2C**, 10 µM **3PP** and 10 µM **2M3PP**. [^{14}C]L-lactate uptake is normalized with respect to the absence of any inhibitor. Removal of the hydroxyl-group (i.e., Y331F) significantly reduces the affinity of the mutated transporter for the inhibitors. Data are represented as mean ± SEM from three (WT) to six (Y331F) independent experiments, each in triplicate. Individual data points are shown as open circles (WT) and squares (Y331F).

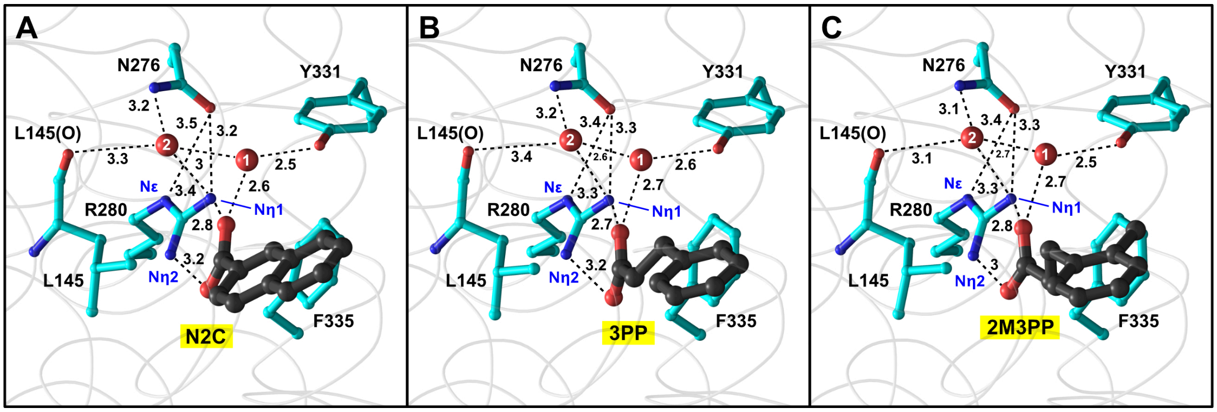

Supplementary Figure 7. Hydrogen-bonding relay network. The role of the two identified water molecules (numbered, red spheres) in the binding of A) **N2C**, B) **3PP** and C) **2M3PP** is shown. The three guanidinium nitrogen atoms of R280 are labelled Nε, Nη1 and Nη2. The backbone carbonyl oxygen of L145 (TM5) is labelled L145(O). The second water molecule (2) interacts with the first water molecule (1), with the amide-group of the side chain of N276 (TM8), with the Nη1 nitrogen of the guanidinium group of R280 (TM8), as well as with the backbone carbonyl oxygen of L145 (L145(O), TM5). Both, L145 (TM1) and N276 (TM8) have been shown to be functionally-important residues.[2] Distances are given in Ångström (Å). PDB IDs of displayed structures are 6ZGS (**3PP**), 6ZGT (**N2C**) and 6ZGU (**2M3PP**).

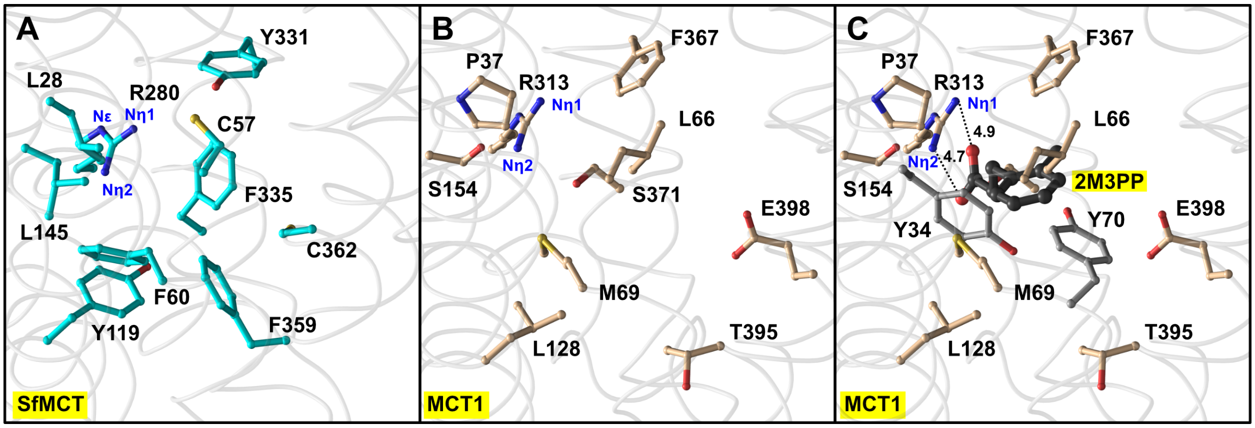

Supplementary Figure 8. Comparison of residues involved in inhibitor binding in SfMCT with corresponding residues in human MCT1 (PDB ID 7CKR). A) SfMCT residues within a distance of 4 Å from bound inhibitors (see Figure 2). B) Corresponding residues in human MCT1. The following residue pairs are shown (SfMCT-*MCT1*): L28-*P37*, C57-*L66*, F60-*M69*, Y119-*L128*, L145-*S154*, R280-*R313*, Y331-*F367*, F335-*S371*, F359-*T395*, C362-*E398*). C) **2M3PP** was placed in the human MCT1 structure by aligning the MCT1 structure with the respective **2M3PP** -bound SfMCT structure. The distance between the Nη atoms of the guanidinium group of R313 and the oxygen atoms of the carboxylate group of **2M3PP** are 4.7 and 4.9 Å, which is significantly larger than observed for the **2M3PP**-bound SfMCT structure (see Figure 2). Y34 (grey), L66 and Y70 (grey) lead to major clashes between MCT1 residues and **2M3PP**.

Supplementary Table 1. Data collection, processing and refinement statistics

**Data collection and processing**	**1OHN2C**[a]	**N2C**[b]	**2M3PP**[c]	**3PP**[d]
PDB ID	6ZGR	6ZGT	6ZGU	6ZGS
Beamline	X06SA, Swiss Light Source - SLS	X06SA, Swiss Light Source - SLS	X06SA, Swiss Light Source - SLS	X06SA, Swiss Light Source - SLS
Detector	Eiger 16M	Eiger 16M	Eiger 16M	Eiger 16M
Wavelentgh (Å)	1.0	1.0	1.0	1.0
Space group	$P2_12_12$	$P2_12_12$	$P2_12_12$	$P2_12_12$
Unit-cell: a, b, c (Å); $\alpha = \beta = \gamma$ (°)	103.6, 199.6, 62.5; 90	102.5, 199.6, 61.9; 90	102.1, 199.7, 61.6; 90	102.2, 199.7, 61.6
Anisotropy directions[e]				
overall (Å)	**2.67**	**2.39**	**2.41**	**2.39**
along h axis (Å)	2.88	2.57	2.65	2.66
along k axis (Å)	2.46	2.23	2.18	2.15
along l axis (Å)	3.34	3.02	2.94	2.86
Resolution (Å)[f]	47.14-2.46 (2.63-2.46)	49.66-2.23 (2.43-2.23)	49.44-2.18 (2.38-2.18)	49.48-2.15 (2.39-2.15)
Measured reflections	1,409,036 (54,588)	2,464,345 (102,888)	1,255,551 (52,969)	783,613 (32,991)
Unique reflections	30,629 (1,360)	42,191 (2,111)	41,475 (1,828)	41,154 (2,050)
Redundancy	46.0 (40.1)	58.4 (48.7)	30.3 (29.0)	19.0 (16.1)
R_{meas}[g]	0.11 (4.0)	0.12 (3.6)	0.13 (2.8)	0.07 (2.0)
$R_{p.i.m.}$[h]	0.02 (0.6)	0.02 (0.5)	0.03 (0.5)	0.02 (0.5)
$CC_{1/2}$[i]	1.0 (0.6)	1.0 (0.7)	1.0 (0.7)	1.0 (0.7)
Mean $I/\sigma(I)$	34.4 (1.6)	43.0 (1.6)	30.3 (1.7)	32.0 (1.8)
Completeness (%)[k]	94.3 (94.0)	94.8 (80.9)	94.4 (82.6)	94.4 (82.0)
**Refinement**				
Resolution (Å)	14.98–2.46	19.96–2.23	14.97–2.18	14.97–2.15
R_{work}/R_{free}[l] (%)	22.09/25.50	21.53/23.99	21.55/24.45	21.60/25.25
No. of atoms	5779	5822	5822	5819
Protein	5749	5790	5790	5790
Ligands	28	26	24	22
Waters	2	6	8	7
Mean B factor (Å^2)	102.4	101.3	97.0	89.4
Protein	102.6	101.4	97.1	89.5
Ligands	80.4	71.8	73.6	63.9
RMSD				
Bond length (Å)	0.009	0.009	0.009	0.008
Bond angle (°)	1.191	1.097	1.105	1.125
Ramachandran plot (%)				
Favoured region	98.8	98.2	98.2	98.6
Allowed region	100	100	100	100
Disallowed region	0	0	0	0

[a] Datasets from 5 crystals were merged; in the deposited PDB-file **1OHN2C** is abbreviated 1HN.
[b] Datasets from 7 crystals were merged; in the deposited PDB-file **N2C** is abbreviated FIV.
[c] Datasets from 3 crystals were merged; in the deposited PDB-file **2M3PP** is abbreviated 02Q.
[d] Datasets from 2 crystals were merged.
[e] The anisotropic resolution limits were computed with AIMLESS[3] based on $CC_{1/2} > 0.50$.
[f] These statistics are for data that was truncated by STARANISO software (http://staraniso.globalphasing.org/) to remove poorly measured reflections affected by anisotropy. Values in parentheses are for the highest resolution shell.
[g] R_{meas} as defined by Diederichs and Karplus (1997)[4].
[h] Precision-indicating merging R factor $R_{p.i.m}$ as defined by Weiss (2001)[5].
[i] $CC_{1/2}$ is the Pearson correlation coefficient of two-half data sets as described by Karplus and Diederichs (2012)[6].
[k] The completeness after the anisotropic correction was obtained by least-square fitting an ellipsoid to the reciprocal lattice points at the cut-off surface defined by a local mean $I/\sigma I$ threshold of 1.2, rejecting outliers in the fit due to spurious deviations, and calculating the fraction of observed data lying inside the ellipsoid.
[l] Random 5% reflections from working set were excluded from refinement for R_{free} calculation.

References

1. Núñez, M. F. *et al.* Transport of L-lactate, D-lactate, and glycolate by the LldP and GlcA membrane carriers of *Escherichia coli*. *Biochem. Biophys. Res. Commun.* **290**, 824–829 (2002).

2. Bosshart, P. D., Kalbermatter, D., Bonetti, S. & Fotiadis, D. Mechanistic basis of L-lactate transport in the SLC16 solute carrier family. *Nat. Commun.* **10**, 2649 (2019).

3. Evans, P. R. & Murshudov, G. N. How good are my data and what is the resolution? *Acta Crystallogr. Sect. D Biol. Crystallogr.* **69**, 1204–1214 (2013).

4. Diederichs, K. & Karplus, P. A. Improved R-factors for diffraction data analysis in macromolecular crystallography. *Nat. Struct. Biol.* **4**, 269–275 (1997).

5. Weiss, M. S. Global indicators of X-ray data quality. *J. Appl. Crystallogr.* **34**, 130–135 (2001).

6. Karplus, P. A. & Diederichs, K. Linking crystallographic model and data quality. *Science* **336**, 1030–1034 (2012).
