# Supplementary material for: The making of a potent L-lactate transport inhibitor
Source: Commun Chem. 2021 Sep 6;4:128. doi: 10.1038/s42004-021-00564-5 (PMC9814091; doi:10.1038/s42004-021-00564-5)
Supplement: Supplementary file 2 — Reporting Summary [file 42004_2021_564_MOESM2_ESM.pdf]

nature research

Corresponding author(s): Prof. Dr. Dimitrios Fotiadis

Last updated by author(s): Aug 3, 2021

Reporting Summary

Nature Research wishes to improve the reproducibility of the work that we publish. This form provides structure for consistency and transparency in reporting. For further information on Nature Research policies, see our Editorial Policies and the Editorial Policy Checklist.

Statistics

For all statistical analyses, confirm that the following items are present in the figure legend, table legend, main text, or Methods section.

n/a	Confirmed	
☐	☒	The exact sample size (*n*) for each experimental group/condition, given as a discrete number and unit of measurement
☐	☒	A statement on whether measurements were taken from distinct samples or whether the same sample was measured repeatedly
☒	☐	The statistical test(s) used AND whether they are one- or two-sided<br>*Only common tests should be described solely by name; describe more complex techniques in the Methods section.*
☒	☐	A description of all covariates tested
☒	☐	A description of any assumptions or corrections, such as tests of normality and adjustment for multiple comparisons
☒	☐	A full description of the statistical parameters including central tendency (e.g. means) or other basic estimates (e.g. regression coefficient) AND variation (e.g. standard deviation) or associated estimates of uncertainty (e.g. confidence intervals)
☒	☐	For null hypothesis testing, the test statistic (e.g. *F*, *t*, *r*) with confidence intervals, effect sizes, degrees of freedom and *P* value noted<br>*Give P values as exact values whenever suitable.*
☒	☐	For Bayesian analysis, information on the choice of priors and Markov chain Monte Carlo settings
☒	☐	For hierarchical and complex designs, identification of the appropriate level for tests and full reporting of outcomes
☒	☐	Estimates of effect sizes (e.g. Cohen's *d*, Pearson's *r*), indicating how they were calculated

Our web collection on statistics for biologists contains articles on many of the points above.

Software and code

Policy information about availability of computer code

Data collection	X-ray diffraction datasets were collected at the X06SA (PXI) beamline of the Swiss Light Source (Paul Scherrer Institute, Villigen, Switzerland) using<br>an EIGER 16M detector (Dectris).
Data analysis	Structure determination:<br>The datasets were indexed and integrated with XDS and then merged using BLEND of the CCP4 program suite without truncation of the resolution. Scaling and averaging of symmetry-related intensities for all datasets were performed by aP_scale with truncation of the data at the best high-resolution along h, k or l axis determined by AIMLESS. Due to the anisotropic nature of the diffraction data, the STARANISO software (http://staraniso.globalphasing.org/) was applied. This program performs an anisotropic cut-off of merged intensity data to perform Bayesian estimation of structure amplitudes and to apply an anisotropic correction to the data. The structures were obtained by molecular replacement with Phaser using the SfMCT structure (PDB ID 6G9X) or the N2C structure (i.e., 2-naphthoate (N2C)-bound SfMCT structure) as search model. The final structures were obtained after multiple rounds of model building with Coot and refinement with phenix.refine. The following settings were used for all refinement runs: XYZ coordinates, individual B-factors, occupancies and TLS strategies. Figures involving structures were prepared using PyMol (The PyMol Molecular Graphics System; Schrödinger).<br><br>Analysis of transport assays:<br>Data of transport assays were analyzed using Prism 6.

For manuscripts utilizing custom algorithms or software that are central to the research but not yet described in published literature, software must be made available to editors and reviewers. We strongly encourage code deposition in a community repository (e.g. GitHub). See the Nature Research guidelines for submitting code & software for further information.

Data

Policy information about availability of data

All manuscripts must include a data availability statement. This statement should provide the following information, where applicable:

- Accession codes, unique identifiers, or web links for publicly available datasets
- A list of figures that have associated raw data
- A description of any restrictions on data availability

Atomic models have been deposited in the Protein Data Bank under accession numbers 6ZGR, 6ZGS, 6ZGTand 6ZGU.

Field-specific reporting

Please select the one below that is the best fit for your research. If you are not sure, read the appropriate sections before making your selection.

☒ Life sciences ☐ Behavioural & social sciences ☐ Ecological, evolutionary & environmental sciences

For a reference copy of the document with all sections, see nature.com/documents/nr-reporting-summary-flat.pdf

Life sciences study design

All studies must disclose on these points even when the disclosure is negative.

Sample size	For transport studies, presented data originate from three to six independent experiments, each performed in triplicates.
Data exclusions	In transport studies, no data was excluded from the analysis.
Replication	Transport experiments were independently repeated at least 3 times.<br>For X-ray crystallography experiments more >1000 crystals were measured.
Randomization	Randomization is not necessary and was thus not applied.
Blinding	Blinding was not applied because it is not necessary in the field of X-ray crystallography.

Reporting for specific materials, systems and methods

We require information from authors about some types of materials, experimental systems and methods used in many studies. Here, indicate whether each material, system or method listed is relevant to your study. If you are not sure if a list item applies to your research, read the appropriate section before selecting a response.

Materials & experimental systems

n/a	Involved in the study	
☒	☐	Antibodies
☒	☐	Eukaryotic cell lines
☒	☐	Palaeontology and archaeology
☒	☐	Animals and other organisms
☒	☐	Human research participants
☒	☐	Clinical data
☒	☐	Dual use research of concern

Methods

n/a	Involved in the study	
☒	☐	ChIP-seq
☒	☐	Flow cytometry
☒	☐	MRI-based neuroimaging
